# Transcriptomic analysis of coxsackievirus B3 infection in induced pluripotent stem cell-derived brain-like endothelial cells

**DOI:** 10.1128/jvi.01824-24

**Published:** 2024-12-13

**Authors:** Sarah F. Hathcock, Julia Mamana, Taryn E. Keyzer, Nadine Vollmuth, Mohammad-Reza Shokri, Henry D. Mauser, Robert N. Correll, Daryl W. Lam, Brandon J. Kim, Jon Sin

**Affiliations:** 1 Department of Biological Sciences, The University of Alabama 164491 , Tuscaloosa, Alabama, USA; 2 Center for Convergent Biosciences and Medicine, The University of Alabama 8063 , Tuscaloosa, Alabama, USA; 3 Department of Microbiology, Heersink School of Medicine, The University of Alabama at Birmingham 155591 , Birmingham, Alabama, USA; 4 Alabama Life Research Institute, The University of Alabama 8063 , Tuscaloosa, Alabama, USA; University of Michigan Medical School, Ann Arbor, Michigan, USA

**Keywords:** coxsackievirus B3, blood-brain barrier, brain endothelial cells, RNA sequencing, induced pluripotent stem cells

## Abstract

Viral aseptic meningitis is a neuroinflammatory condition that occurs when viruses gain access to the central nervous system (CNS) and induce inflammation. The blood–brain barrier (BBB) is comprised of brain endothelial cells (BECs) that stringently regulate the passage of molecules, toxins, and pathogens from the circulation into the CNS. Through their unique properties, such as complex tight junctions, reduced rates of endocytosis, expression of efflux transporters, and restricted expression of leukocyte adhesion molecules, the BBB is often able to limit pathogen entry into the brain; however, certain neurotropic pathogens, such as coxsackievirus B3 (CVB3) are able to infect the CNS. We have previously demonstrated that CVB3 can infect and disrupt induced pluripotent stem cell-derived brain-like endothelial cells (iBECs), but the host response to this infection remains unknown. Here, we investigate global host transcriptional changes during CVB3 infection of iBECs using RNA sequencing. We validated our data set by exploring pathways altered by CVB3 using quantitative real-time PCR (qPCR) and enzyme-linked immunosorbent assay of upregulated cytokines and interferon signaling molecules.

**IMPORTANCE:**

Coxsackievirus B3 (CVB3) is a leading cause of viral aseptic meningitis that can produce severe disease in susceptible individuals. To gain access to the central nervous system, CVB3 must cross central nervous system barriers, such as the blood–brain barrier. Previously, we have shown that CVB3 infects a human stem cell-derived brain-like endothelial cell model. Here, we report the global transcriptome of stem cell-derived brain-like endothelial cells to CVB3 infection and provide proof-of-concept validation of the dataset using molecular biology techniques. These data could inform novel mechanisms of CVB3-mediated blood–brain barrier dysfunction.

## INTRODUCTION

As a part of the *Enterovirus* genus and the *Picornaviridae* family, coxsackievirus B3 (CVB3) is a single-stranded, non-enveloped RNA virus that is a common human pathogen (1). CVB3 is a typically self-resolving virus known most commonly to cause mild, nonspecific, sub-clinical infections, but in rare cases can lead to significant and potentially fatal systemic disease, especially in young children and infants (2–6). These diseases include myocarditis, pancreatitis, and meningitis (7–9). Viral aseptic meningitis is a serious condition occurring when viruses, such as CVB3, interact with and penetrate the blood–brain barrier (BBB), subsequently causing inflammation in the central nervous system (CNS) (10, 11). It is thought that up to 95% of viral aseptic meningitis cases can be attributed to enteroviruses such as coxsackieviruses (12–15). Additionally, long-term neurological defects, such as impairment in neuronal development, seizure disorders, Alzheimer’s disease, and schizophrenia have been suggested to be long-term sequelae of CVB3 infection (16–20). Even though there is a clear and significant disease burden associated with CVB3, there is limited understanding of the host’s BBB response to disease (10).

The BBB is comprised of highly specialized brain endothelial cells (BECs) that maintain CNS homeostasis by regulating the passage of molecules while restricting pathogens such as CVB3. The BECs of the BBB are unique, possessing complex tight junctions and high transendothelial electrical resistance (TEER) relative to peripheral endothelial cells (21, 22). While there may be other entry routes into the CNS, we have previously demonstrated that CVB3 can infect and disrupt BECs and potentially establish persistent infection in these cells (10). It is known that CVB3 can profoundly alter cell signaling following infection, resulting in the activation of antiviral pathways and interferon responses, as well as the disruption of host cell translation in the liver, heart, and neonatal CNS, specifically in neurons and astrocytes (18, 23–26). However, little is known about the host response of other members of the CNS, such as BECs, to CVB3 infection. Here, we utilize an induced pluripotent stem cell (iPSC)-derived brain-like endothelial cell (iBEC) model and RNA sequencing (RNA-seq) to discover the global transcriptome during CVB3 infection of BECs as others have found utility in understanding host–pathogen interactions using iPSC-derived BBB models in conjunction with transcriptomics (10, 27–30). We have previously demonstrated that this model provides utility in understanding CVB3-mediated BBB dysfunction, and we hypothesize that this model will respond to viral infection by altering the host transcriptome (10). We found that CVB3 induces a strong interferon response and cytokine production at 5 days post-infection (PI). Additionally, we discovered that signaling pathways associated with CVB3 infection are highly enriched, prompting us to determine that the manipulation of one particular mitogen activated protein kinase (MAPK) pathway modulates viral infection in iBECs. Finally, cytokine expression was validated by quantitative PCR (qPCR). For the first time, we present a transcriptomic analysis of human iBECs during CVB3 infection, and interpretation of this validated data set could uncover novel physiological alterations that occur at the BBB following CVB3 exposure.

## MATERIALS AND METHODS

### Maintenance of iPSCs and differentiation of iBECs

IMR90-C4 (WiCell) iPSCs were maintained and passaged as previously described (22, 31, 32). Briefly, cells were maintained on six-well plates coated with Matrigel (Corning; 354234) and passaged at 80% confluency using EDTA (Thermo Fisher; 15040). StemFlex (Thermo Fisher; A3349401) media were changed on cells daily. iBEC generation was conducted as previously described (22, 31, 32). Briefly, iPSCs were incubated with Accutase (StemCell Technologies; 7920), counted, and seeded in Matrigel coated 75 cm^2^ flasks at 10,000 cells/cm^2^ in StemFlex + 10 µM ROCK inhibitor (Y-27632 dichloride) (Tocris Biosciences; 1254). iPSCs were expanded in StemFlex media for 3 days before initiating iBEC differentiation in unconditioned media (Dulbecco’s modified Eagle medium [DMEM] – F12 medium 1:1 [Gibco; 11330], 20% knockout serum replacement [Gibco; 10828028], 1% minimal essential medium [Gibco; 11140050], 0.5% GlutaMAX [Gibco; 35050], and 0.07% beta-mercaptoethanol [G Biosciences; BC98]) for 6 days with daily media changes. Cells were then changed into endothelial cell (EC) media (human endothelial cell serum-free medium [Gibco; 11111-044]) containing 1% B27 (Gibco; 17504044), 20 ng/mL basic fibroblast growth factor (bFGF) (Peprotech; 100-18B), and 10 µM retinoic acid (RA) (Sigma-Aldrich; 2625) (EC +/+) for 2 days. Differentiated iBECs were then purified onto a collagen-IV (Sigma-Aldrich; C5533) and fibronectin (Sigma-Aldrich; F1141) matrix in EC +/+ at 500,000 cells/cm^2^ in 24-well and 48-well plates or 1 × 10^6^ cells/cm^2^ in 12-well transwells (Corning; 3460). Media were changed to EC without bFGF or RA (EC −/−) the day following purification, and TEER was read on transwells to validate successful purification. iBECs were maintained in EC −/− for downstream applications, and validation using immunofluorescence was performed 2 days following purification. iBECs 2 days post-purification express markers including glucose transporter 1 (GLUT1) (Fig. S1A), claudin-5 (Fig. S1B), occludin (Fig. S1C), zonula occludens-1 (ZO-1) (Fig. S1D), cluster of differentiation 31 (CD31) (Fig. S1E), vascular-endothelial cadherin (VE-cadherin) (Fig. S1F), and P-glycoprotein (P-gp) (Fig. S1G).

### Generation of CVB3 virus

The pMKS1 plasmid that was used to generate CVB3 stocks was prepared as described previously (33). This plasmid was a generous gift from Dr. Ralph Feuer (San Diego State University, CA, USA). pMKS1 contains the backbone of the myocarditic Nancy H3 variant of CVB3 (pH3) and was modified to include a unique SfiI restriction site allowing for insertion of foreign fragments of DNA. Gene sequences for enhanced green fluorescent protein (eGFP) were amplified from plasmids with sequence-specific primers to add SfiI recognition sequences. PCR products were then cloned into the pMKS1 plasmid to generate the eGFP–CVB3 construct. The eGFP–CVB3 plasmid was then linearized by digesting with ClaI restriction enzyme (New England BioLabs; R0197S). Linearized products were then subsequently used for *in vitro* transcription with the mMESSAGE mMACHINE T7 kit (Thermo Fisher; AM1344). HeLa cells were then transfected with viral transcripts using Lipofectamine 2000 (Thermo Fisher; 11668019). Once 50% of transfected cells expressed viral eGFP, cells were scraped, subjected to three freeze–thaw cycles, and centrifuged at 2,000 rpm to remove excess cellular debris. The resultant supernatant is “passage 1 virus”. For viral expansion, “passage 1 virus” was placed onto a second set of HeLa cells that were harvested once these cells also exhibited 50% viral eGFP expression. This supernatant of the second set of samples is “passage 2 virus” and was subsequently titered *via* plaque assay and used for downstream experiments. Viral propagation did not exceed “passage 2”.

### Infection of iBECs

iBECs were infected at a multiplicity of infection (MOI) of 10 at 2 days following purification (see above) for 2 or 5 days for all experiments detailed. MOI was calculated using the number of iBECs seeded. Frozen stocks of virus were used to inoculate cells, whereas mock infected cells received an equivalent volume of DMEM (Sigma-Aldrich; D6429) containing 10% fetal bovine serum (Corning; 35010CV). For all experiments, vehicle conditions were dimethyl sulfoxide (DMSO) (Amresco; 0231) at the same volume as the treatment group unless otherwise specified.

### Lactate dehydrogenase viability assay

The cytotoxicity of CVB3 infection and/or U0126 treatment in iBECs was measured using the LDH-Cytox Assay Kit according to the manufacturer’s instructions (Biolegend; 426401). iBECs were grown in a 24-well plate and infected as previously described at an MOI of 10 for 2 or 5 days (10). Lysis control wells were collected alongside infected samples. At 30 min prior to the end of either infection timepoint, 50 µL of lysis buffer was added to the lysis control wells, and the plate was replaced in the incubator at 37°C + 5% CO_2_. After the full infection time, 100 µL of cell media was transferred from the respective wells of the 24-well plate to a 96-well plate. Then, 100 µL of working solution was added to the 96-well plate, which was then incubated at 37°C + 5% CO_2_ for 30 min. Then, 50 µL of stop solution was added to each well, and a SpectraMax iD3 Microplate Reader (Molecular Devices, San Jose, CA, USA) was used to determine the absorbance of each well at 490 nm. Cell cytotoxicity is expressed as a percent of the lysis control absorbance.

### Plaque assays

iBECs were infected, and plaque assays were conducted as previously described (10). Cell media were collected from iBECs at 5 days PI and underwent four freeze–thaw cycles (to account for non-lytic, extracellular microvesicle-bound virus) before being serially diluted in unconditioned DMEM (Sigma-Aldrich; D6429) (1). The dilutions were added to HeLa cells grown to confluency in six-well plates. The plates were rocked every 15 min for 1 h before covering cells with a 0.6% agarose (Invitrogen; 16500) and DMEM (Gibco; 12800–082) mixture. The agarose–DMEM plug was left on the cells for 48 h, and a fixative solution of 25% acetic acid (Spectrum; A110) and 75% methanol (Sigma-Aldrich; 34860–4L-R) was gently added on top of the plug for 20 min. The solution and agarose–DMEM plugs were removed, and crystal violet solution (0.6% crystal violet [Sigma-Aldrich; C0775], 79.4% ethanol, 20% sterile water) was added to each well for 1 h before being rinsed off with tap water. Once the plates were dry, plaques were counted. Data are expressed as PFU per mL.

### Microscopy

Cells were imaged with the Nikon Ti2 inverted epifluorescence microscope equipped with a Qi2 camera (Nikon, Tokyo, Japan) using NiS Elements software version AR.5.30.05. Images were taken with a 700-ms exposure time for basal focus monolayer images, and a FITC green channel was used to visualize eGFP with a 1-s exposure time. All images were taken at an Analog of 1. Analysis of images was completed with NIH ImageJ software (FIJI).

### RNA extraction and sequencing

RNA was extracted from iBECs at 2 or 5 days PI using the Macherey–Nagel NucleoSpin RNA kit (Macherey–Nagel; 740955) according to manufacturer’s protocol. Standard RNA sequencing on RNA isolates and library preparation using pol(A) enrichment were performed by Azenta US, Inc using the Illumina NovaSeq 6000 platform with 2 × 150 base pair paired-end sequencing. Fastq formatted files were uploaded to the University of Alabama Supercomputer Cluster (UAHPC) and were matched with the sequencing facility’s md5sum files. Raw reads were assessed for quality using FastQC version 0.11.5 (34). Raw reads were cleaned of adapter contamination, and nucleotides with poor quality scores (Phred < 30) were removed *via* TRIMGALORE version 0.4.2 (www.bioinformatics.babraham.ac.uk/projects/trim_galore). FastQC was subsequently run on the cleaned data files to assess improvement. Reads were mapped to the annotated *Homo sapiens* genome (GRCh38.110) using STAR version 2.5.3a (35). Gene counts were quantified by featureCounts through the Subread package version 2.0.1 (36). The count data were analyzed for differential expression using DESeq2 version 1.34.0 through R version 4.1.2 (37, 38). Ensembl ID values were cross-referenced with gene names using the Annotables version 0.191 R library (39). Separate heatmaps and volcano plots were constructed for the 2-day experiment and 5-day experiment using the R libraries ComplexHeatmap and ggplot2 for values with an adjusted *P* ≤ 0.05 and an absolute log_2_ fold change of >2 or >1, respectively (40–42).

### PathfindR enrichment analyses

All pathway enrichment analyses were conducted using R version 4.3.3 and RStudio version 2023.12.1+ 402 (38). Enriched terms were calculated based on adjusted *P*-values of ≤0.05 and a log_2_ fold change of >2. Enriched terms were determined in separated analyses from Kyoto encyclopedia of genes and genomes (KEGG), gene ontology: cellular components (GO-CC), gene ontology: molecular function (GO-MF), gene ontology: biological processes (GO-BP), and Reactome databases using pathfindR (43–45). Volcano plots, enriched term visualizations, and heat maps were generated using R, ggplot2, ComplexHeatmap, and pathfindR (38, 40–42, 45). Protein–protein interaction networks were constructed using Cytoscape version 3.10.2 and Search tool for retrieval of interacting genes/proteins (STRING) database (46, 47).

### Immunofluorescence

iBECs were seeded in 48 wells for each marker visualized as previously described (22, 31, 32). iBECs were fixed in ice-cold 100% methanol (VWR BDH Chemicals; 1135) for 15 min at room temperature and incubated with primary antibodies in blocking buffer (10% fetal bovine serum [Gibco; A56694-01] in PBS) at their respective dilutions overnight at 4°C. The following day, primary antibodies were aspirated, and cells were washed three times with phosphate-buffered saline (PBS). Cells were then incubated with secondary antibody 488 anti-mouse (Thermo Fisher; A11001) at a 1:200 dilution in blocking buffer at room temperature protected from light. Secondary antibodies were aspirated, and cells were then incubated with 4′,6-diamidino-2-phenylindole (DAPI) (Promokine; PK-CA707-40043) at 1:5,000 in PBS for 15 min at room temperature protected from light. Stained cells were then imaged using the Nikon Ti2 microscope as described above and analyzed using ImageJ software. Primary antibodies used include anti-GLUT-1 (Thermo Fisher; MA5-11315 [SPM-498]) (1:100), anti-Claudin-5 (Thermo Fisher; 35-2500 [4C3C2]) (1:50), anti-occludin (Thermo Fisher; 33-1500 [OC-3F10]) (1:200), anti-ZO-1 (Thermo Fisher; 33-9100 [ZO-1A12]) (1:100), anti-CD31 (Thermo Fisher; PA5-16301) (1:25), anti-VE-cadherin (Santa Cruz; SC-52752 [BV9]) (1:25), anti-P-gp (Thermo Fisher; MA5-13854 [F4]) (1:25) (22).

### Western blotting

Cell lysates were collected with radioimmunoprecipitation assay (RIPA) buffer (140 mM NaCl, 1% Triton X-100, 0.1% sodium deoxycholate [SDS], 0.1% sodium dodecyl sulfate, 0.5 mM ethylene glycol bis(2-aminoethyl ether)-N,N,N′,N′ tetraacetic acid [EGTA]) with a protease inhibitor cocktail (Thermo Fisher; 78429) and phosphatase inhibitor (Roche; 04 906 837 001). Samples were passed through a 27-gauge needle 10 times and centrifuged at 15,000×*g* for 10 min at 4°C to remove excess cellular debris. A bicinchoninic acid (BCA) assay (Thermo Fisher; 23227) was performed to standardize protein levels through dilution with molecular water. Equal amounts of sample buffer (Morganville Scientific; LB0100) with β-mercaptoethanol were added to each sample followed by denaturation *via* boiling at 100°C. Equal amounts of lysate were loaded into 4%–12% Bis-Tris gels (Thermo Fisher; NW04125BOX) and later transferred onto nitrocellulose membranes (Amersham; GE10600003). Ponceau S stain was conducted to verify successful transfer and equal loading, and membranes were blocked in 3% bovine serum albumin (BSA) (Fisher; BP1605) in Tris-buffered saline plus 0.1% Tween-20 (TBST) for 1 h at room temperature. Following blocking, membranes were washed three times for 5 min each with TBST and incubated in 3% BSA in TBST plus primary antibodies overnight at 4^°^C while shaking. The following day, membranes were washed three times for 5 min each with TBST and incubated in 3% BSA in TBST with appropriate horseradish peroxidase (HRP) conjugated secondary antibodies (Jackson Laboratories; 102646-160, anti-mouse; 102645-182, anti-rabbit) for 1 h at room temperature. Membranes were again washed for 5 min three times in TBST and imaged using enhanced chemiluminescent substrate (Thermo Fisher; 1859697) and iBright FL1500 (Thermo Fisher, Waltham, MA, USA), or Azure 300 (Azure Biosystems, Dublin, CA, USA) imaging software. Densitometry of blots is normalized to Ponceau S stain or total extracellular signal-regulated kinase 1/2 (ERK1/2) protein and expressed as fold change compared with uninfected, vehicle-treated controls. Primary antibodies used include anti-coxsackievirus B (Megadiagnost; M47) (1:1,000), anti-ERK (Cell Signaling Technologies; 4696 [L34F12]) (1:2,000), anti-phospho-ERK1/2 (pThr202 pTyr204) (Cell Signaling Technologies; 9101) (1:1000), anti-β-tubulin (Cell Signaling Technologies; 2128 [9F3]) (1:1,000).

### Quantitative real-time PCR (qPCR)

iBECs were seeded in 24 wells and infected with vehicle or CVB3 as described above. RNA was collected and extracted at 5 days PI using the Macherey–Nagel NucleoSpin (Macherey–Nagel; 740955) and NucleoSpin RNA Plus kit (Macherey–Nagel; 740984). RNA quantities were determined using a Nanodrop 1000. cDNA was generated using normalized RNA amounts and the QScript First Strand cDNA Synthesis kit (Quantabio; 95047) and diluted 1:10 in nuclease-free water. SYBR Green (Applied Biosystems; A25743) qPCR was then conducted on samples using *GAPDH* as a housekeeping gene and a Quantstudio 3 (Thermo Fisher, Waltham, MA, USA). Results were calculated using the delta–delta Ct method and represented as fold change versus *GAPDH*. The following primers were utilized: *IFIT1* forward 5′-ggaatacacaacctactagcc-3′; *IFIT1* reverse 5′-ccaggtcaccagactcctca-3′, *IFIT2* forward 5′-gggaaactatgcctgggtc-3′; *IFIT2* reverse 5′-ccttcgctctttcattttggtttc-3′, *IFIT3* forward 5′-tgaggaagggtggacacaactgaa-3′; *IFIT3* reverse 5′-aggagaattctgggttgttgggct-3′, *OAS3* forward 5′-tgctgccagcctttgacgcc-3′; *OAS3* reverse 5′-tcgcccgcattgctgtagctg-3′, *MX1* forward 5′-aggaccatcggaatcttgac-3′; *MX1* reverse 5′-tcaggtggaacacgaggttc-3′, *ISG15* forward 5′-gagaggcagcgaactcatct-3′; *ISG15* reverse 5′-cttcagctctgacaccgaca-3′ (48–50).

### Enzyme-linked immunosorbent assay

PBS, wash buffer, reagent diluent, and substrate solution were prepared according to the R&D Systems Human RANTES/CCL5 DuoSet ELISA product (R&D Systems; DY278-05) datasheet. iBECs were seeded in 24 wells and infected with vehicle or CVB3 as previously described, and cell media were collected 5 days PI. Reconstituted capture antibody was incubated overnight in a 96-well plate at room temperature. The wells were aspirated, washed three times with wash buffer, and blocked for 1 h with reagent diluent. The aspiration and washes were repeated as described above. Standards were diluted in the plate with reagent diluent, and collected media samples were added to the appropriate wells. Standards and samples were allowed to incubate for 2 h before the plate was aspirated and washed. Reconstituted detection antibody was diluted in reagent diluent and added to each well, then the plate was incubated for 2 h. Wells were then aspirated and washed as previously described. Streptavidin-HRP was added to each well and incubated for 1 h. The aspiration and washes were repeated as described before substrate solution was added to the wells. The plate was covered and allowed to incubate for 30 min. Stop solution (2N sulfuric acid) was then added to each well, and the optical density was determined at 450 nm (and 570 nm for wavelength correction) using a Spectramax iD3 plate reader (Molecular Devices, San Jose, CA, USA). The concentration (pg/mL) of RANTES/CCL5 in each sample was then determined. The minimum detection limit of this assay is reported at 1.7 pg/mL.

### Statistics

**Fig 1:**
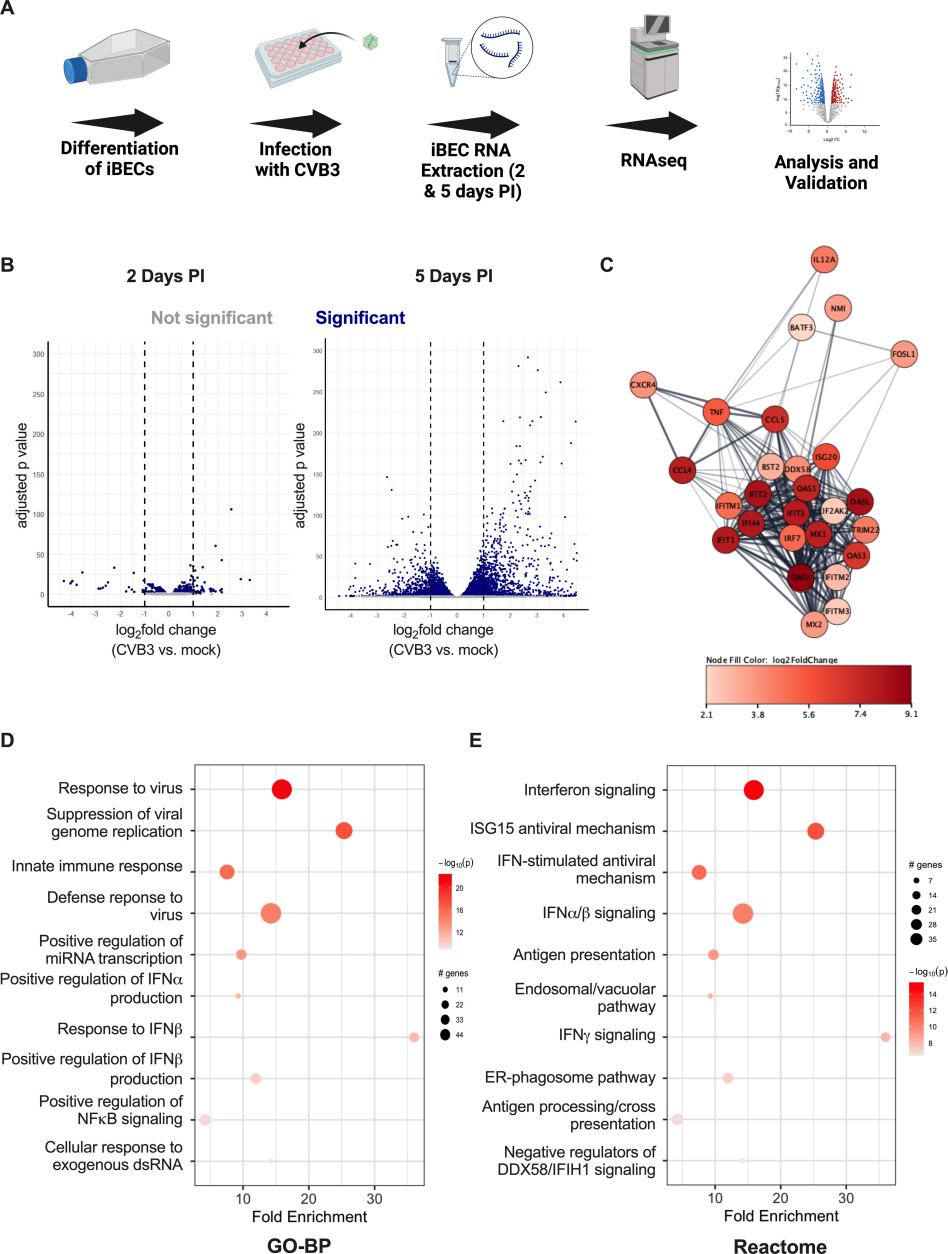
iBECs respond to CVB3 infection. (**A**) Schematic illustrating the complete process of iBEC differentiation, CVB3 infection, RNA sequencing, and subsequent analysis and validation of results. (Created with Biorender.com.) (**B**) Volcano plots representing differentially expressed genes following CVB3 infection at 2 (left) and 5 days PI (right). (**C**) Bubble plot generated from the enriched genes under Reactome’s “response to virus” pathway at 5 days PI. Top 10 enriched pathways following pathfindR pathway enrichment analyses through the (**D**) GO-BP and (**E**) Reactome databases. Experiments were performed in technical triplicate (*n* = 3).

**Fig 2:**
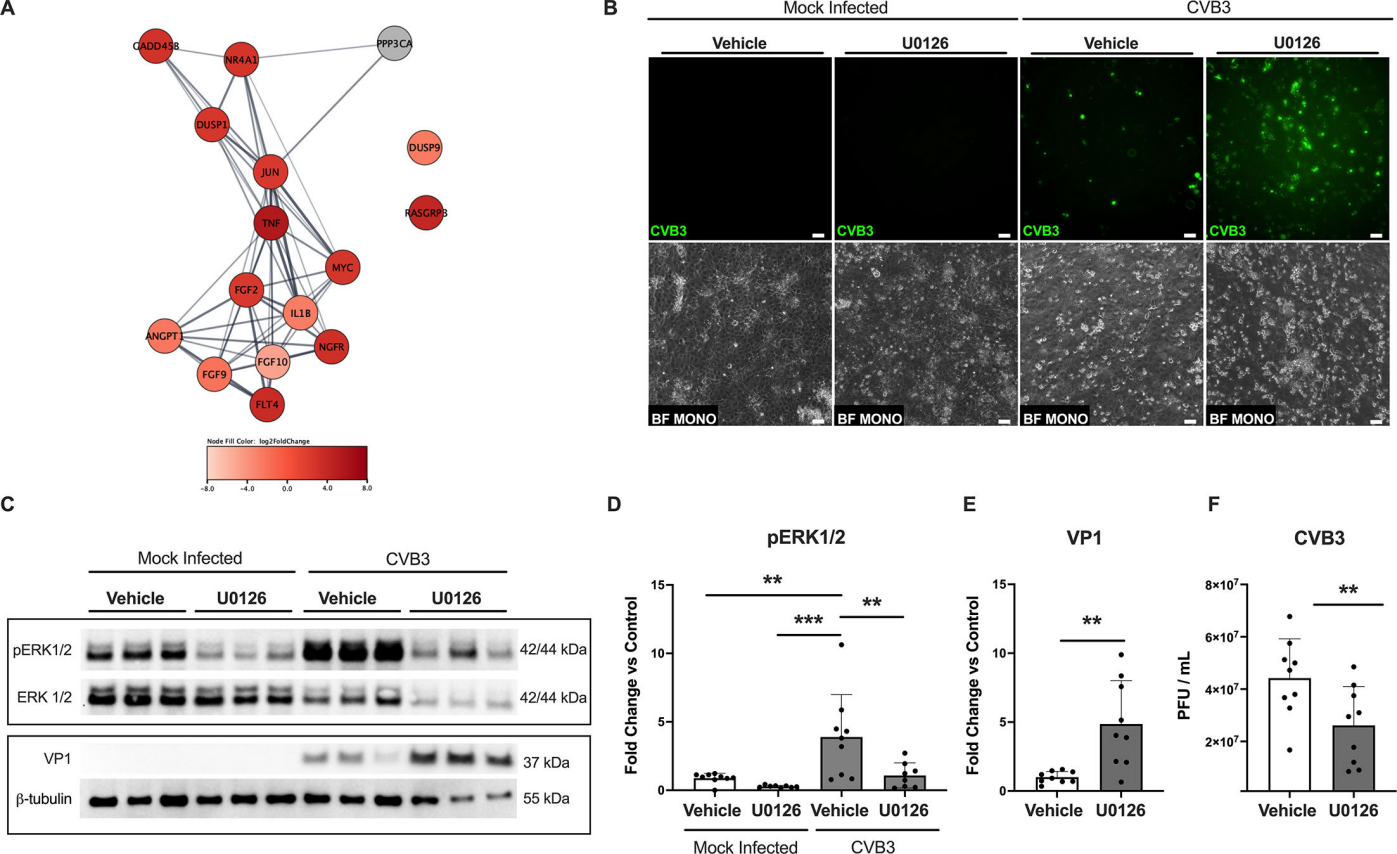
CVB3 activates the MAPK pathway in iBECs. (**A**) Bubble plot generated from enriched genes under KEGG for “MAPK signaling pathway” at 5 days PI. (**B**) Fluorescence microscopy images (top row) visualizing viral eGFP. The second row is phase contrast images of the same field taken concurrently. Scale bars represent 50 µm. (**C**) Western blot detecting total ERK1/2, pERK1/2, VP1, and loading control β-tubulin in iBEC lysates collected 5 days PI that were either infected with CVB3 at MOI 10 or mock infected with equivalent volume DMEM and pretreated with 10 µM U0126 or equivalent volume of DMSO for 1 hour prior to infection. Total ERK serves as a loading control for pERK, while β-tubulin serves as a loading control for VP1. pERK and VP1 blots are boxed to represent separate biological replicates. (**D**) Densitometric quantification of pERK, (**E**) intracellular VP1. (**F**) Quantification of extracellular released virus *via* plaque assay. Baseline inoculum is 1 × 10^6^ PFU. Experiments were performed in technical triplicate with three distinct differentiations (*n* = 8 or 9). Error bars represent standard deviation. A one-way ANOVA with *post hoc* Dunnett’s multiple comparisons (Fig. 2D) or a student’s *T* test (Fig. 2E and F) was performed; ** *P* ≤ 0.01, *** *P* ≤ 0.001; no annotation indicates no significant difference.

Statistical analyses and figure generation were conducted using GraphPad Prism version 10.2.3. For comparison between two groups, a Student’s *t* test was used, and for multiple comparisons, a one-way analysis of variance (ANOVA) with *post hoc* Dunnett’s multiple comparisons was conducted. Statistical tests to determine outliers were performed using a ROUT test and a Q of 1%. Outliers include Fig. S1H CVB3 Vehicle: 44.479%; Fig. 1D Mock U0126 0.745, CVB3 U0126 5.330; Fig. 2B *OAS3* Mock: 1.894; Fig. 2C *ISG15* CVB3: 602.111; Fig. 2E *IFIT2* CVB3: 2307.938, 2942.82; Fig. 2F *IFIT3* CVB3: 1618.237, 1242.326 (all fold change versus *GAPDH*). Fig. 2G CVB3: 56.72998 pg/mL. Statistical significance was accepted for *P* < 0.05.

**Fig 3:**
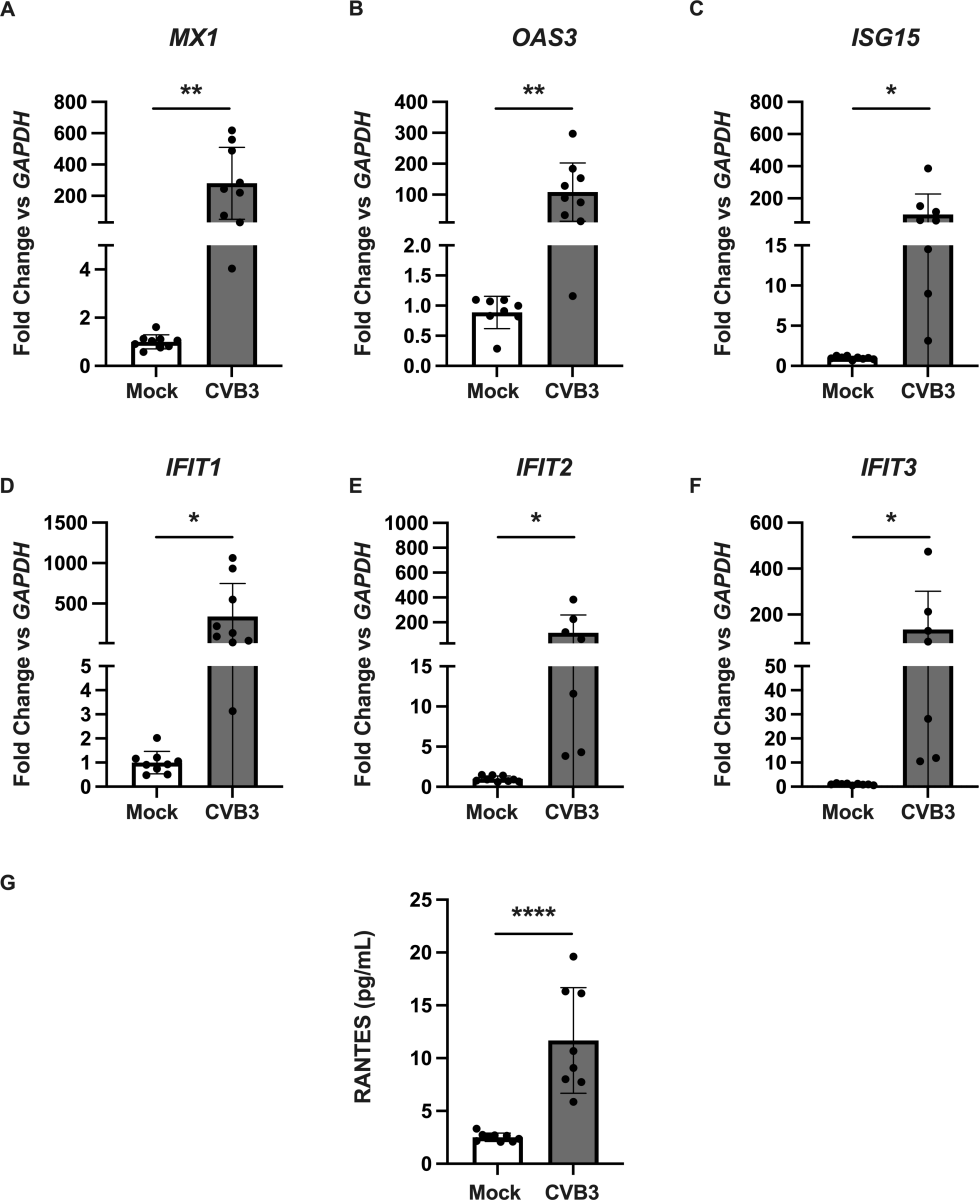
CVB3 activates antiviral mechanisms. Real-time qPCR identifying fold change compared with *GAPDH* of various antiviral genes in mock-infected and CVB3-infected iBECs for (**A**) *MX1*, (**B**) *OAS3*, (**C**) *ISG15*, (**D**) *IFIT1*, (**E**) *IFIT2*, and (**F**) *IFIT3* at 5 days PI. (**G**) ELISA detecting extracellular concentration of RANTES/CCL5 in media collected at 5 days PI from mock-infected cells and CVB3-infected cells. Experiments were performed in technical triplicate with three distinct differentiations (*n* = 7–9). Error bars represent standard deviation. Student’s *t* test was performed; * *P* ≤ 0.05, ** *P* ≤ 0.01, **** *P* ≤ 0.0001.

## RESULTS

### iBECs transcriptionally upregulate antiviral and interferon programs upon CVB3 infection

To discover the global host transcriptomic changes that occur in iBECs during CVB3 infection, iBECs were infected with CVB3 at an MOI of 10 and RNA-seq, as well as subsequent DESeq analysis was conducted at 2 and 5 days PI (Fig. 1A). Robust infection was found to begin at 2 days PI as indicated by a 10-fold increase in PFU (relative to initial inoculum) in our previous studies (10). Additionally, at 2 days PI, barrier function remains intact, with minimal tight junction disruption and no significant change in TEER (10). At 5 days PI, robust infection and viral shedding remains, tight junctions are disrupted, and TEER is significantly decreased; however, cell viability is maintained (Fig. S1H and I). This makes these timepoints suitable for comparing prolonged infection (10). At 2 days PI, there were relatively few differentially expressed transcripts compared with 5 days PI in relation to time-matched mock-infected controls (Fig. 1B). A threshold of transcripts differentially expressed with an adjusted *P*-value of ≤0.05 and an absolute log_2_fold change of >2 was used for further analysis. With this threshold, only 19 transcripts were significantly differentially expressed at 2 days PI, seven of which were upregulated and 12 that were downregulated (Table 1; Fig. S2A). These transcripts commonly were involved in the inhibition of viral replication and the activation of antiviral and anti-apoptotic transcripts, including those encoding interleukins, interferon regulatory factors, and interferon-induced proteins, indicating activation of type I and III interferon signaling (48, 51, 52). At 5 days PI, 681 transcripts were significantly differentially expressed, with 487 upregulated and 194 downregulated (Fig. 1B; Fig. S3). Because of the stark increase in the quantity of differentially expressed transcripts at 5 days PI, the remainder of the study focuses on this timepoint. From these transcripts, a pathway enrichment analysis was conducted using the program pathfindR to understand the alteration of signaling pathways and biological functions during infection (45). The top enriched pathway includes a complex network of antiviral and cytokine signaling that is represented through protein–protein interactions characterized by cross-referencing the enriched terms with the search tool for retrieval of interacting genes/proteins (STRING) database (Fig. 1C). The use of STRING in understanding the transcriptomic data allowed for an overarching, systematic view of the interactions between the enriched processes and altered transcripts we observed following CVB3 infection (47). Notably, some of the interacting transcripts represented in this network include interferon-induced protein with tetratricopeptide repeats 1–3 (IFIT1, IFIT2, and IFIT3), interferon-induced transmembrane proteins 1–3 (IFITM1, IFITM2, and IFITM3), 2′−5′ oligoadenylate synthases (OAS1, OAS2, and OAS3), and Myxovirus resistance proteins (MX1 and MX2) (53–55). Some of these transcripts experience drastic upregulation when compared to mock-infected controls as soon as 2 days PI (*IFIT1* log_2_fold change = 2.156, adjusted *P* = 0.002; *IFIT2* log_2_fold change = 1.615, *P* = 0.002; *IFIT3* log_2_fold change = 1.764, adjusted *P* = 1.90 E-5), indicating the progressive activation of antiviral signaling over time.

**TABLE 1:** Differentially expressed genes in iBECs at 2 days PI

Gene	Log_2_(fold change)	Adjusted *P* value
*TNFRSF1B*	−3.545654	4.87E-28
*CGB3*	−3.962215	2.51E-17
*ARRDC3*	2.562055	4.96E-107
*EGR1*	2.163707	1.26E-43
*CGA*	−4.316182	3.05E-17
*CYP19A1*	−2.246059	2.37E-34
*DUSP15*	2.941364	1.1E-19
*MX1*	2.104064	0.0000185
*SLC16A14*	3.311939	1.13E-18
*IFIT1*	2.155535	0.00197
*CGB5*	−4.041941	1.01E-14
*DIO3*	−2.899	5.83E-08
*ZNF442*	2.15938	0.000000156
*CGB8*	−2.820711	2.64E-08
*ERVW-1*	−2.604252	1.89E-11
*ERVFRD-1*	−3.985696	4.45E-17
*LCMT1-AS2*	−2.502587	9.2E-16
ENSG00000267943	−3.790611	7.39E-13
*ERVV-2*	−2.71233	5.83E-09

The top 200 differentially expressed transcripts at 5 days PI are represented in a heatmap, clustering upregulated transcripts for immune responses, such as IFIT and IFITM proteins (*IFIT1-3, IFITM1-3*) (*IFITM1* log_2_fold change = 4.854, adjusted *P* < 2.225074 E-308; *IFITM2* log_2_fold change = 2.766, adjusted *P* = 1.58 E-135; *IFITM3* log_2_fold change = 2.404, adjusted *P* = 2.47 E-163) as well as pro-inflammatory cytokines (*TNF* log_2_fold change = 5.386, adjusted *P* = 7.15 E-52*; CXCL1* log_2_fold change = 2.567*,* adjusted *P* = 5.68 E-10*; CXCL2* log_2_fold change = 2.740*,* adjusted *P* = 3.89 E-11*; IL6* log_2_fold change = 4.660, adjusted *P* = 2.82 E-51) (Fig. S3) (56, 57). Pathway enrichment analyses of the data set at 5 days PI using pathfindR and the GO-BP database revealed the top enriched biological process to be involved in “response to virus” (Fig. 1D), and a variety of pathways including analysis with the Reactome, GO-BP, GO-CC, and KEGG databases show signaling and cellular functions associated with general antiviral responses, such as type I interferon (alpha and beta) signaling as well as CVB3-specific responses, such as miRNA production (Fig. 3D and E; Fig. S2B through F; Table 2) (25, 58–62). Taken together, these data suggest that iBECs globally respond to CVB3 infection and alter their transcriptome, particularly in regard to antiviral and inflammatory signaling. Additionally, these data demonstrate that there are more differentially expressed transcripts at 5 days PI than at 2 days PI.

**TABLE 2:** Enriched pathways in iBECs at 5 days PI

	Term description	Fold enrichment
GO-MF		
GO:0042605	Peptide antigen binding	13.5010331
GO:0004842	Ubiquitin-protein transferase activity	2.340468
GO:0001228	DNA-binding transcription activator activity, RNA polymerase II-specific	2.4091063
GO:0000981	DNA-binding transcription factor activity, RNA polymerase II-specific	4.9919786
GO:0003700	DNA-binding transcription factor activity	2.527741
GO:0000976	Transcription cis-regulatory region binding	2.8054095
GO:0003725	Double-stranded RNA binding	8.1209221
GO:0044389	Ubiquitin-like protein ligase binding	10.8915897
GO:0001227	DNA-binding transcription repressor activity, RNA polymerase II-specific	2.7718117
GO:1990782	Protein tyrosine kinase binding	3.0859504
Reactome		
R-HSA-913531	Interferon signaling	12.0853723
R-HSA-1169408	ISG15 antiviral mechanism	7.6091928
R-HSA-1169410	Antiviral mechanism by IFN-stimulated genes	9.1435568
R-HSA-909733	Interferon alpha/beta signaling	22.8275784
R-HSA-983170	Antigen presentation: folding, assembly and peptide loading of class I MHC	15.9618125
R-HSA-1236977	Endosomal/vacuolar pathway	27.0020661
R-HSA-877300	Interferon gamma signaling	12.8034113
R-HSA-1236974	ER-phagosome pathway	5.5954046
R-HSA-1236975	Antigen processing-cross presentation	5.2402932
R-HSA-936440	Negative regulators of DDX58/IFIH1 signaling	10.5804014
KEGG		
HSA05170	Human immunodeficiency virus one infection	3.6305299
HSA04668	TNF signaling pathway	9.5914675
HSA05169	Epstein–Barr virus infection	5.5547107
HSA04612	Antigen processing and presentation	6.1719008
HSA04621	NOD-like receptor signaling pathway	6.8381856
HSA05163	Human cytomegalovirus infection	4.7742282
HSA05164	Influenza A	6.2997213
HSA04064	NF-kappa B signaling pathway	5.3929231
HSA04060	Cytokine-cytokine receptor interaction	5.7462525
HSA05203	Viral carcinogenesis	3.3664914
HSA04010	MAPK signaling pathway	2.5451137
GO-CC		
GO:0030670	Phagocytic vesicle membrane	10.1610562
GO:0055038	Recycling endosome membrane	6.0707221
GO:0031901	Early endosome membrane	4.2948794
GO:0012507	ER to Golgi transport vesicle membrane	7.200551
GO:0000785	Chromatin	2.2235014
GO:0090575	RNA polymerase II transcription regulator complex	4.3396178
GO:0005839	Proteasome core complex	5.4457948
GO:0045121	Membrane raft	1.8224117
GO:0000502	Proteasome complex	2.1529887
GO:0001533	Cornified envelope	5.6450312
GO:0005741	Mitochondrial outer membrane	2.2765208
GO-BP		
GO:0009615	Response to virusokl	15.8973203
GO:0045071	Negative regulation of viral genome replication	25.3488784
GO:0045087	Innate immune response	7.566513
GO:0051607	Defense response to virus	14.2428481
GO:1902895	Positive regulation of miRNA transcription	9.7451066
GO:0032727	Positive regulation of interferon-alpha production	9.2578512
GO:0035456	Response to interferon-beta	36.0027548
GO:0032728	Positive regulation of interferon-beta production	11.9456145
GO:0043123	Positive regulation of I-kappaB kinase/NF-kappaB signaling	4.2081142
GO:0071360	Cellular response to exogenous dsRNA	14.2428481

### CVB3 infection activates MAPK signaling in iBECs

Pathway enrichment analyses determined that MAPK signaling was significantly enriched upon CVB3 infection at 5 days PI as illustrated and further validated in a STRING-based protein–protein interaction network (Fig. 2A). Following general antiviral and immune responses (Fig. 1; Fig. S2), MAPK signaling was one of the prominent enriched processes determined from our pathfindR analysis. MAPK signaling has been tied to controlling viral replication by CVB3 during infection of T cells and cardiac myocytes and is commonly known to modulate inflammatory responses during infection of many cell types, making it an ideal candidate for proof-of-concept validation of the RNA-seq data (62, 63). Furthermore, other positive-sense RNA viruses have shown upregulation of this pathway, including yellow fever virus (64). Increased understanding of this pathway may provide insight into therapeutics to modulate viral replication during infection (65). Transcripts involved in activation and downstream transcription of the p38 and c-Jun N-terminal kinase (JNK) branches of the MAPK signaling cascade are upregulated (*GADD45B* log_2_fold change = 2.860, adjusted *P* = 2.73 E-38); however, the increased prevalence of transcripts relating to the MEK/ERK pathway in our protein interaction network prompted our focus on this branch of MAPK signaling (66, 67). These transcripts include the downregulated dual specificity phosphatase 9, (DUSP9), a specific negative regulator of MEK/ERK signaling (*DUSP9* log_2_fold change = −2.546, adjusted *P* = 2.18 E-33), as well as the upregulated tumor necrosis factor (TNF), an upstream effector of MEK/ERK signaling (*TNF* log_2_fold change = 5.386, adjusted *P* = 7.15 E-52) (63, 68). We also observe upregulation of downstream MEK/ERK transcription factors, such as those forming AP-1 to promote cytokine expression (*JUN* log_2_fold change = 2.305, adjusted *P* = 6.51 E-282) (Fig. 2A) (63, 68). Using the pharmacological MEK inhibitor U0126 to block MEK/ERK signaling, we observed an increase in the number of eGFP-positive CVB3-infected iBECs upon U0126 treatment (Fig. 2B) (53). Furthermore, CVB3 infection resulted in an increased abundance of phosphorylated ERK1/2 (pERK1/2) when compared with uninfected controls, which was rescued in U0126-treated CVB3-infected iBECs 5 days PI (Fig. 1C and D). Additionally, viral capsid protein (VP1) levels in ERK1/2 inhibited cells were increased compared with vehicle-treated infected controls, while rates of cell lysis remained similar among conditions (Fig. 1C through E; Fig. S1I). Interestingly, plaque assays demonstrated decreased viral shedding in U0126 treated cells when compared with vehicle-treated cells despite increased intracellular VP1 levels and increased visualization of viral eGFP in U0126-treated cells (Fig. 2F). Some of these findings indicate that this branch of the MAPK pathway potentially regulates viral release, explaining the increased intracellular VP1 with decreased viral shed. However, the increased presence of viral eGFP in U0126-treated cells requires further investigation. Overall, these data confirm the transcriptomic analysis, suggesting that MAPK pathways are enriched during CVB3 infection and demonstrate that CVB3 activates MAPK signaling to modulate viral replication, serving as a proof-of-concept to validate the RNA-seq data.

### CVB3 infection activates pro-inflammatory and antiviral signaling in iBECs

Our transcriptomic analysis suggests that at 5 days PI, CVB3 activates antiviral signaling in iBECs, demonstrating that iBECs respond to CVB3 infection (Fig. 3B through E). To further confirm this observed activation from our RNA-seq data, we performed qPCR of select highly upregulated transcripts, many of which were indicated in the protein–protein interaction network titled “response to virus” at 5 days PI (Fig. 1C). Previous literature has shown that type I interferon activation stimulates the upregulation of antiviral gene *MX1* following CVB3 and other viral infections, such as Influenza A, in order to limit viral RNA replication (55, 69, 70). We observe a similar increase of *MX1* transcripts in iBECs at 5 days PI in our sequencing data (log_2_fold change = 7.684, adjusted *P* = 5.99 E-78), validated by qPCR (Fig. 3A). The protein encoded by 2′−5′-oligoadenylate synthase (*OAS3*) is a selective sensor for double-stranded RNA (54). Double-stranded RNA is a potent pathogen-associated molecular pattern associated with viral infection and is known to activate RNase L by synthesizing 2′−5′ linked oligoadenylates to cleave viral RNA (54, 71, 72). The upregulation of *OAS3* at 5 days PI (log_2_fold change = 6.774, adjusted *P* = 2.08 E-248) signifies the activation of a specific RNA−antiviral response in iBECs (Fig. 3B). Because transcript levels of *IFIT1, IFIT2,* and *IFIT3* are increased at 5 days PI compared with 2 days PI, we aimed to validate the increase in antiviral signaling at 5 days PI. At this timepoint, transcripts of canonical downstream effectors of type I interferon signaling, such as interferon-stimulated gene 15 kDa (*ISG15)* (log_2_fold change = 7.068, adjusted *P* = 4.07 E-73) (Fig. 3C), as well as the previously mentioned *IFIT* transcripts (*IFIT1)* (Fig. 3D)*, (IFIT2)* (Fig. 3E), and (*IFIT3)* (Fig. 3F) are significantly upregulated *via* qPCR, confirming the transcriptomic analysis (*IFIT1* log_2_fold change = 7.925, adjusted *P* = 4.02 E-13; *IFIT2* log_2_fold change = 8.325, adjusted *P* = 2.49 E-9; *IFIT3* log_2_fold change = 7.884, adjusted *P* = 9.48 E-17) (53, 73). A significant increase in the secretion of chemokine RANTES/CCL5 was also observed in infected cell media when compared with mock-infected cell media, in accordance with our data set (log_2_fold change = 7.001, adjusted *P* = 8.29 E-31) and other meningeal infection data in iBECs, including *Neisseria meningitidis* (Nm) infection (Fig. 3G) (69, 74).

## DISCUSSION

The CNS is protected by specialized barriers, such as the BBB, by controlling the passage of molecules, pathogens, and immune cells into the brain to prevent neuroinflammation (75–77). It has been shown that the BBB is highly selective, restricting entry to approximately 98% of all small molecules and 100% of large molecules (78, 79). The BECs comprising the BBB are unique, possessing complex tight junctions, high TEER values, and forming a dynamic, adaptable neurovascular unit (NVU), interacting with astrocytes, pericytes, neurons, and glial cells (80–82). CVB3 must interact with the BECs of the BBB before entering the brain and infecting the CNS. However, the exact response of the BBB during this interaction remains poorly understood (83–85).

Modeling the BBB during diseases such as meningitis poses unique challenges due to the highly specialized phenotype of BECs (21). *In vitro* models of the BBB have select advantages. Isolated rat brain capillaries are able to respond to co-culture cues with astrocytes and pericytes; however, these models produce limited TEER values and hence increased permeability during co-culture and lack key BBB phenotypes, such as functional efflux transporters in monoculture (21). Human primary brain endothelial cells, although isolated directly from human patients, are often limited in supply and quickly lose their phenotype when in culture, greatly limiting the scalability and increasing the variation between different primary cell lines (21). Immortalized BECs are used often in BBB modeling, including the highly characterized and commonly used human cerebral microvascular endothelial cell D3 (hCMEC/D3) line (30, 86). However, hCMEC/D3 monolayers are unable to differentiate between neurotropic and non-neurotropic viruses (57). Furthermore, we have observed that the hCMEC/D3s are non-permissive to CVB3 (data not shown).

Using iBECs as a model for host–pathogen interactions during meningitis has proven highly beneficial in recent years as these cells are highly scalable and produce, but more importantly retain, a more robust BBB phenotype over time and are able to respond to intercellular cues by other members of the neurovascular unit (NVU), including astrocytes, pericytes, and neurons (87, 88). iBECs have been shown to express high levels of efflux transporters, possess complex tight junctions with a reflected higher and more biologically relevant TEER level, and limit rates of endocytosis, allowing for further recapitulation of the BBB phenotype *in vitro* (31, 32). Additionally, iBECs allow for the targeted study of CVB3 interactions with the BBB, without the confounding effects of pancreatitis or myocarditis, as multi-system infection has been reported in animal models (89). We have demonstrated that iBECs can be used to successfully model CVB3 infection in addition to other meningeal pathogens (10, 28, 30, 90–93). iBECs have utility in modeling these infections due to the biological expression of surface receptors required for microbial interaction and invasion that other cell lines may not possess (90, 92). For example, iBECs constitutively express antiviral transcripts, such as those encoding IFITMs, which are known to be associated with antiviral defense and the restriction of viral membrane fusion; however, their distinct roles in antiviral immunity still remain poorly understood (57, 92, 94, 95). Our RNA-seq data reveals the activation of transcripts encoding these proteins, presenting significant transcriptional upregulation of *IFITM1*, *IFITM2*, and *IFITM3* at 5 days PI.

RNA-seq analyses of iBECs infected with meningeal pathogens have recently been performed for Group B *Streptococcus* (GBS) infection, Nm infection, and SARS-CoV-2 infection, providing valuable insight into mechanisms of invasion and BBB alteration, as well as supporting the validity of the *in vitro* model and its application to human infection (30, 74, 92). These studies revealed global transcriptomic changes in the GBS genome following interaction with iBECs, while Nm infection revealed the upregulation of transcripts associated with cellular stress, inflammation, and hypoxia in iBECs (30, 74). SARS-CoV-2 infection of iBECs revealed an upregulation of type I interferon response and a downregulation of metabolic processes in the host (92). Additionally, multiple groups have conducted RNA-seq following CVB3 infection in neural progenitor cells, murine models of myocarditis, murine astrocytes, HeLa cells, and in mesoderm derived from human iPSCs, revealing an activation of inflammatory responses and the modification of RNA metabolism (29, 56, 96–99). Similarly, we demonstrate a significant change in the global iBEC transcriptome (Fig. 1), the activation of key elements of innate antiviral immunity, and the upregulation of inflammatory responses at 5 days PI (Fig. 3) (10). In contrast, the infection of iBECs with CVB3 also results in different modes of viral production, suggesting an alternate, non-lytic route and the potential for persistent infection (1, 100–103).

As a naked virus, it is widely thought that CVB3 is able to replicate to high viral loads, potentially evading immune response before inducing lytic death, but it has been shown that not all coxsackievirus infections cause enterovirus-typical lytic infection (1, 104–106). We have previously reported that iBECs can maintain productive CVB3 infection and remain viable for unusually long periods of time (up to 9 days PI), which is suggestive of persistent infection (10). In our current study, we observe that CVB3 infection of iBECs triggers a profound immune response in a typically immune-privileged cell type through the upregulation of various inflammatory markers, such as interferons beta and lambda (*IFNB1* log_2_fold change 10.928, adjusted *P* value 1.24 E-18; *IFNL1* log_2_fold change 8.899, adjusted *P* value 1.80 E-62)*,* chemokines, such as RANTES/CCL5 (*CCL5;* log_2_fold change 7.001, adjusted *P* value 8.29 E-31), and cytokines, such as tumor necrosis factor alpha (TNFα) (*TNF*; log_2_fold change 5.386, adjusted *P* value 7.15 E-52). Notably, these responses occur in a seemingly non-lytic manner as evidenced by consistent levels of lactate dehydrogenase release (Fig. S1H and I). Furthermore, these data are in accordance with previous studies stating that CVB3 infection of the neonatal CNS induces the upregulation of these specific markers as well as other inflammatory cytokines and chemokines, which has been validated in animal models through an observed recruitment of immune cells into the CNS (24, 85). Though these responses are typically associated with viral suppression, they may also potentially contribute to the establishment of prolonged infection, differing from lytic infection observed in HeLa cells, HL-1 cardiomyocytes, and other cell types due to disruption of monolayers and death of cells within shorter timepoints (104–106). These responses, along with the low levels of cell lysis following longer timepoints of infection, may render the iBEC model a more useful model to study prolonged infection (10, 107).

Using pathway enrichment analysis on our transcriptomic data set, processes associated with general viral clearance as well as CVB3-specific pathogenesis were upregulated in accordance with previous studies, further validating the use of this infection model (Fig. S2; Fig. S3) (1, 26, 58–60, 108, 109). Additionally, we demonstrate an increase in transcripts for key elements of interferon signaling as well as an increase in RANTES/CCL5 secretion at 5 days PI (Fig. 3G). To observe MAPK signaling, we examined the highly conserved downstream ERK pathway, specifically pERK1/2 versus total ERK1/2, in various conditions. Previous literature has found that enteroviruses and coxsackieviruses activate the MAPK signaling pathway in accordance with our results as we found increased pERK1/2 abundance in iBECs infected with CVB3 compared with mock-infected cells (Fig. 2) (62, 110–113). Further inspection of MAPK signaling during infection revealed that inhibiting ERK1/2 signaling increased the presence of viral eGFP, as well as the abundance of viral protein in cell lysates (Fig. 1B and C E). Interestingly, however, we also observed decreased levels of extracellular viral release in U0126-treated cells (Fig. 2F). The phenomenon of increased invasion with decreased viral shedding could indicate that the ERK MAPK pathway is vital for viral spread, indicating it as a potential target for pharmacological therapeutics of viral meningitis. However, some of the findings presented here are in contrast with previous literature stating that CVB3 viral release and VP1 abundance is decreased following ERK1/2 inhibition in permissive cells (111). Although we determined that CVB3 viral release is attenuated following ERK1/2 inhibition, intracellular VP1 levels are increased (Fig. 1E and F). This could in part be due to varying levels of viral resistance by different cell lines as mentioned previously (10, 32). However, further investigation is required to draw any definitive conclusions. Furthermore, the length of pathway inhibition and infection time may be responsible for these differences, as the contrasting data were observed at earlier timepoints of 7 h PI in HeLa cells rather than 5 days PI in iBECs (111). The advantage of using longer infection timepoints in iBECs when compared with other cell types and BBB models highlights the utility of the iBEC model in studying viral aseptic meningitis as the disease often progresses undetected for days at a time in the clinical setting (114, 115). While this study of the MAPK signaling pathway is a proof-of-concept for the RNA-seq and transcriptomic data, this highlights how little is known about the mechanism through which neurotropic viruses are able to invade the BBB and progress through the brain microenvironment. While our work is focused solely on iBECs, stem cell technologies allow for the future generation of a more complete NVU using stem cell-derived models in an isogenic system (87, 88, 93). This more contextualized unit could improve the understanding of viral pathogenesis at the BBB and in the CNS (88, 90).

One potential pitfall of this study is that the iBECs utilized were differentiated from iPSCs of a female donor. Previous work has suggested that the immune response to CVB3 may be sex-specific, which is evidenced by males being significantly more susceptible to CVB3-induced myocarditis than females (115, 116). Recent CVB3 myocarditis research has found that enhanced expression of inflammatory pathways was more prevalent in males compared with mitochondrial control pathways being enriched in females (98). It remains somewhat unclear whether these types of discrepancies in disease manifestation may be due to immunological differences, differential mitochondrial regulation, or natural genetic variation between individuals (23, 117–120) . To investigate if this difference in immune response occurs during CVB3 meningitis, future studies could include the use of iBECs derived from male-donor iPSCs, such as the DF19-9-11T iPSC line, to allow for a comparison of the differential expression or secretion of inflammatory markers (22, 32). However, whether these aspects translate to this iPSC-based model is yet to be determined.

The findings presented here provide a deeper understanding of transcriptomic changes occurring during CVB3 mediated meningitis. We found that CVB3 induces changes in iBEC transcripts, especially at 5 days PI. The enrichment of antiviral, inflammatory, and MAPK-related transcripts was further validated *via* qPCR, ELISA, Western blotting, and quantification of extracellular virus in line with many previous studies. Overall, these data will serve as a valuable data set in understanding the host–pathogen interactions occurring during viral aseptic meningitis and will be used to further novel discoveries of CVB3-mediated BBB failure during infection.

### Scope

Coxsackievirus B3 (CVB3) is a leading cause of viral aseptic meningitis, proving especially fatal in younger demographics, with very little known about the true mechanism of the disease progression at the blood–brain barrier (BBB). Due to the complex nature of the BBB, the use of an induced pluripotent stem cell-derived brain-like endothelial cell model that produces a robust expression of the BBB phenotype allows for a novel inspection of CVB3 meningitis on a molecular level. Here, we present a robust study using RNA sequencing on a model of CVB3 infection of the BBB to understand the foundational host–pathogen interactions occurring during viral aseptic meningitis that will serve high utility in future study.

## Data Availability

The data sets supporting the conclusions of this article are included within the article and its supplemental files and in the NCBI Gene Expression Omnibus (GEO) repository (121, 122). The data are accessible through GEO Series accession number GSE269413.

## SUPPLEMENTAL MATERIAL

Figure S1 jvi.01824-24-s0001.pdf Validation of successful iBEC differentiation and viability.

Figure S2 jvi.01824-24-s0002.tiff iBECs activate key host signaling pathways following CVB3 infection.

Figure S3 jvi.01824-24-s0003.pdf Top differentially expressed genes.

Supplemental legends jvi.01824-24-s0004.docx Legends for Fig. S1 to S3.

ASM does not own the copyrights to Supplemental Material that may be linked to, or accessed through, an article. The authors have granted ASM a non-exclusive, world-wide license to publish the Supplemental Material files. Please contact the corresponding author directly for reuse.
