# Supplementary figures and images for: Transcriptomic analysis of coxsackievirus B3 infection in induced pluripotent stem cell-derived brain-like endothelial cells
Source: J Virol. 2024 Dec 13;99(1):e01824-24. doi: 10.1128/jvi.01824-24 (PMC11784093; doi:10.1128/jvi.01824-24)

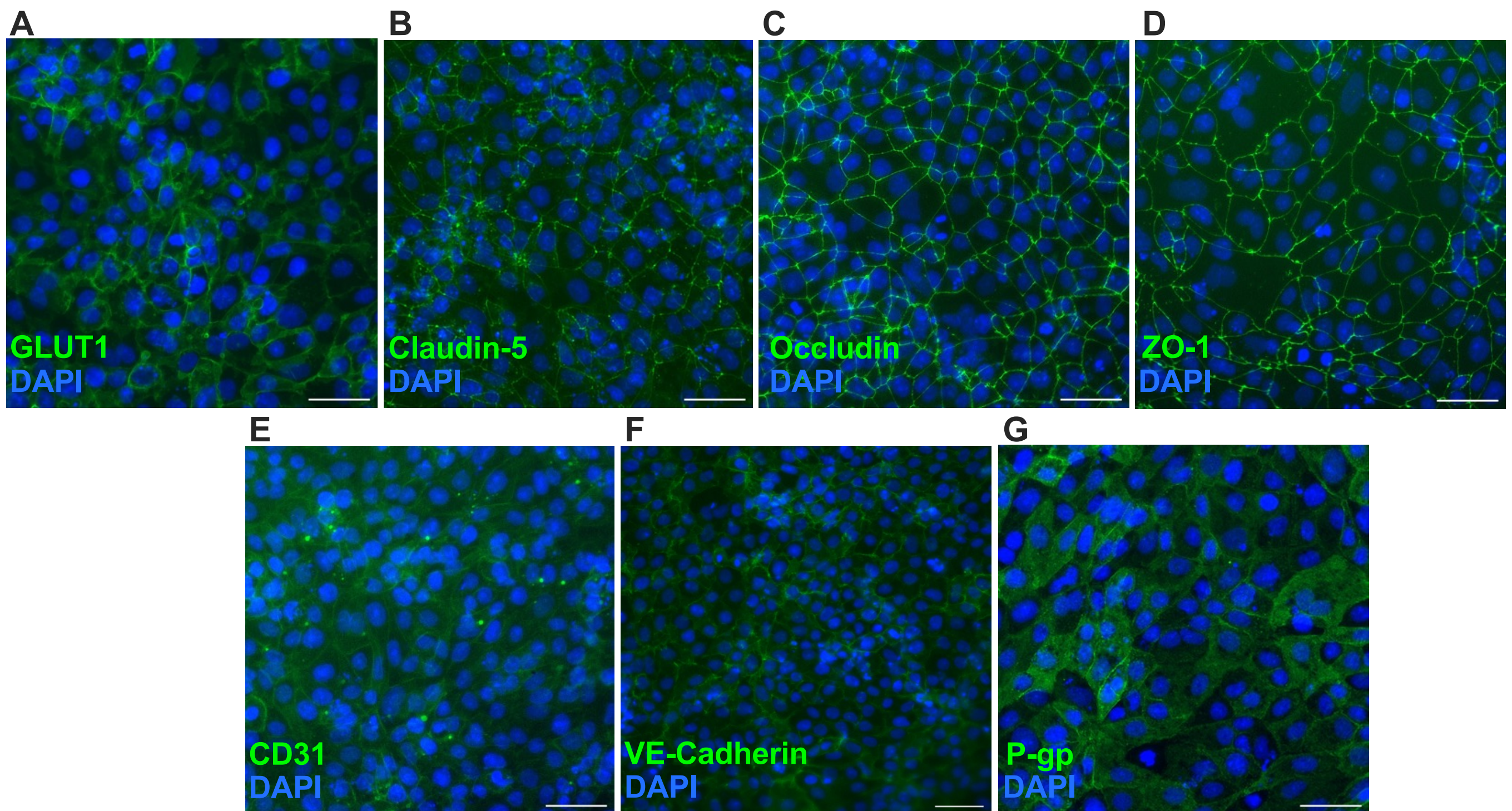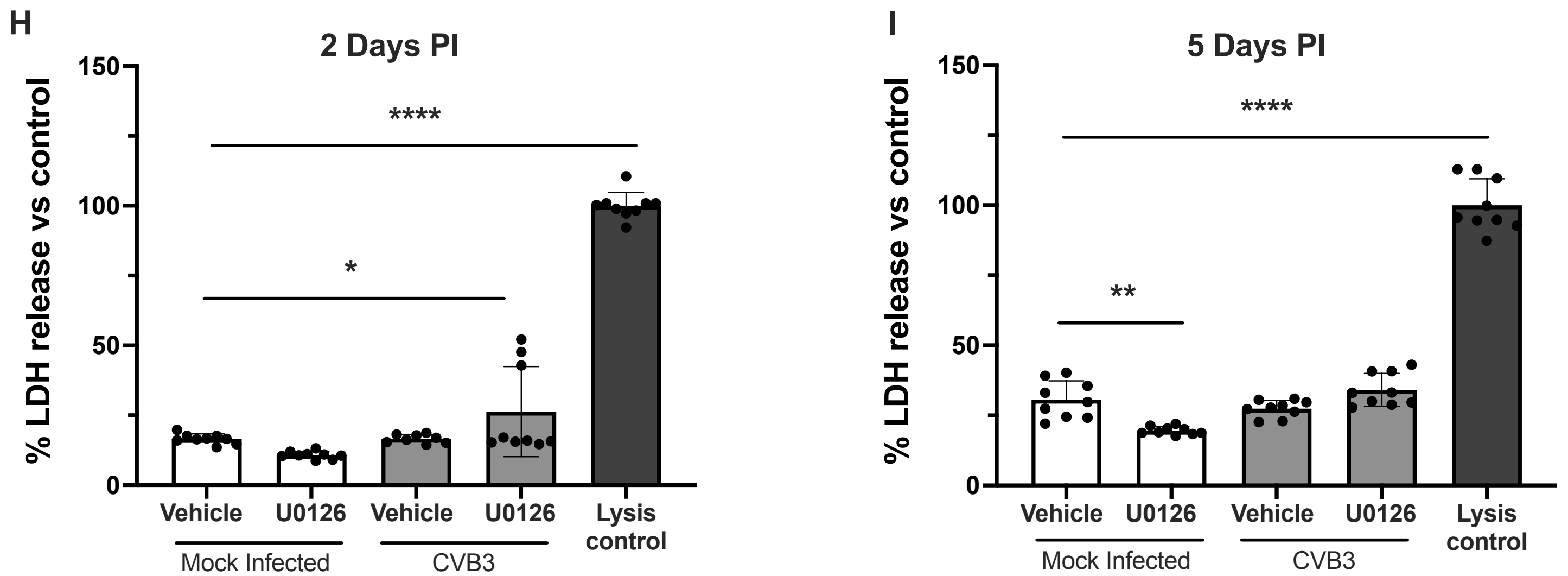

Supplement: Figure S1 — Validation of successful iBEC differentiation and viability. [file jvi.01824-24-s0001.pdf]

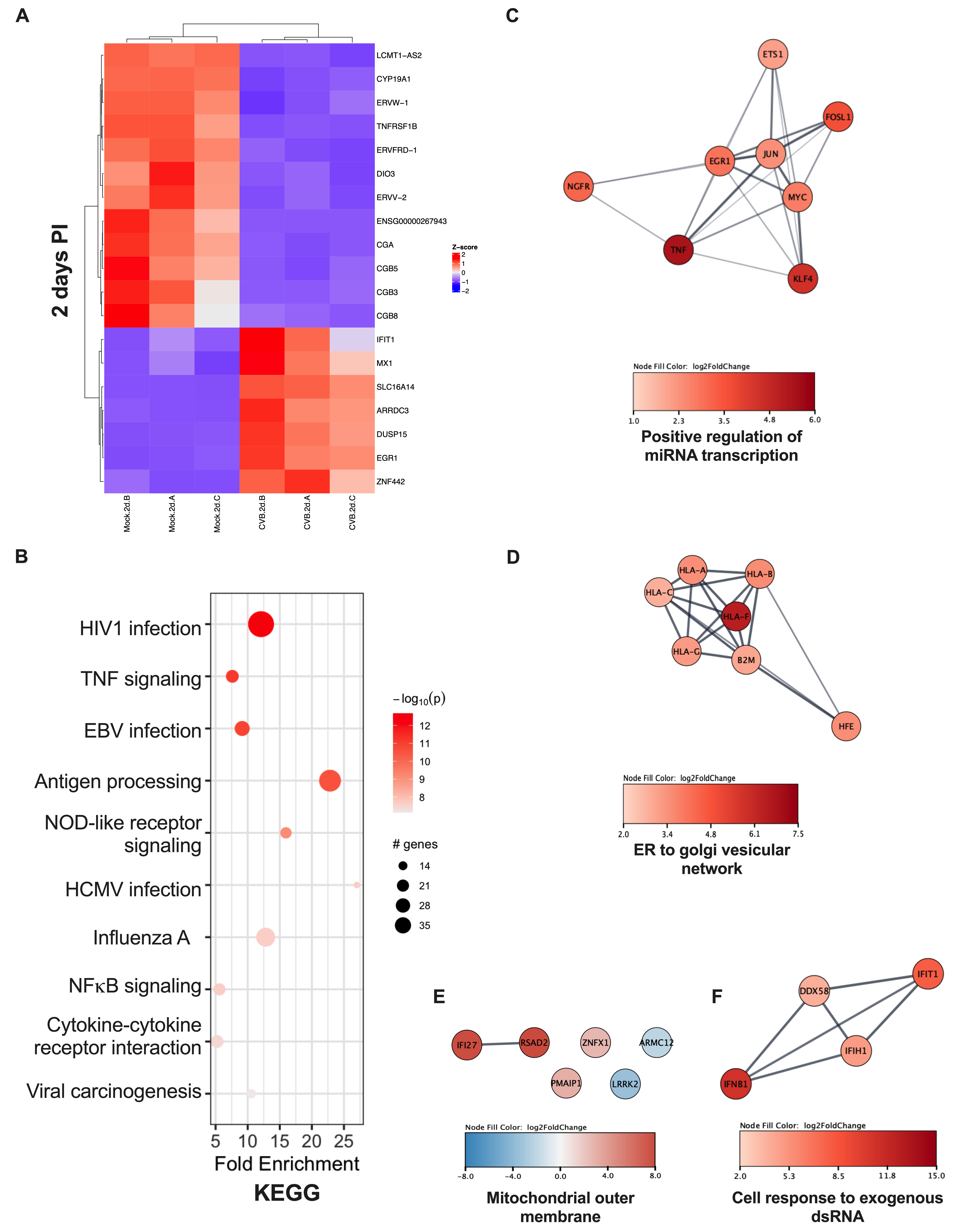

Supplement: Figure S2 — iBECs activate key host signaling pathways following CVB3 infection. [file jvi.01824-24-s0002.tiff]

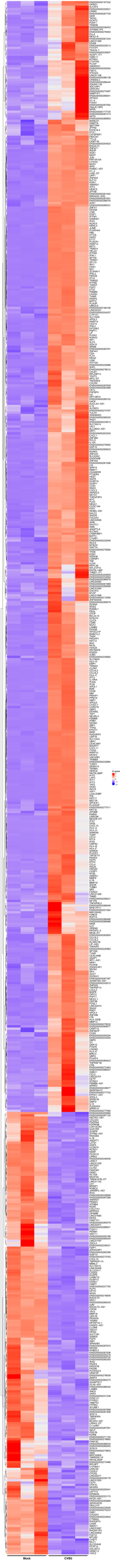

Supplement: Figure S3 — Top differentially expressed genes. [file jvi.01824-24-s0003.pdf]
