# Supplementary material for: Transcriptomic analysis of coxsackievirus B3 infection in induced pluripotent stem cell-derived brain-like endothelial cells
Source: J Virol. 2024 Dec 13;99(1):e01824-24. doi: 10.1128/jvi.01824-24 (PMC11784093; doi:10.1128/jvi.01824-24)
Supplement: Supplemental legends — Legends for Fig. S1 to S3. [file jvi.01824-24-s0004.docx]

**Additional Files**

**Additional File 1: Figure S1**

Validation of successful iBEC differentiation and viability. Representative images of differentiated iBECs immunostained for markers validating a brain endothelial cell phenotype including **(A)** GLUT1, **(B)** Claudin-5, **(C)** Occludin, **(D)** ZO-1, **(E)** CD31, **(F)** VE-Cadherin, and **(G)** P-gp. Scale bar represents 50 µm. Cell viability measured using a lactate dehydrogenase assay, expressed as a percent of lysis control for **(H)** 2 days PI and **(I)** 5 days PI. Viability assays were performed in technical triplicate with 3 distinct differentiations (n = 8 or 9). Error bars represent standard deviation. A one-way ANOVA with *post hoc* Dunnett’s multiple comparisons **(Figure S1H-I)** was performed; * p$\leq$ 0.05, ** p$\leq$ 0.01, **** p < 0.0001; no annotation indicates no significant difference.

**Additional File 2: Figure S2**

iBECs activate key host signaling pathways following CVB3 infection. **(A)** Heatmap representing the 19 upregulated genes with a threshold of p ≤ 0.05 and an absolute log_2_fold change of > 2 at 2 days PI. **(B)** Top 10 enriched pathways following pathfindR pathway enrichment analyses through the KEGG database. **(C)** Bubble plot generated from the enriched genes under GO-BP’s “Positive regulation of miRNA transcription” pathway, **(D)** GO-CC’s “ER to Golgi vesicular network”, **(E)** GO-CC’s “Mitochondrial outer membrane”, and **(F)** GO-BP’s “Cell response to exogenous dsRNA” pathways at 5 days PI. Experiments were performed in technical triplicate (n = 3).

**Additional File 3: Figure S3**

Top differentially expressed genes. Heatmap representing the top 200 differentially expressed genes in iBECs at 5 days PI based on RNA sequencing and DESeq analysis, with their respective Z-scores. Experiments were performed in technical triplicate (n = 3).
